# Advanced Materials in Polymer Electrolyte Fuel Cells

**DOI:** 10.3390/ma10101163

**Published:** 2017-10-10

**Authors:** David Sebastián, Vincenzo Baglio

**Affiliations:** 1 Istituto di Tecnologie Avanzate per l’Energia “Nicola Giordano”, CNR, Via Salita S. Lucia sopra Contesse 5, 98126 Messina, Italy; 2 Instituto de Carboquímica, CSIC, Miguel Luesma Castán 4, 50018 Zaragoza, Spain

## 1. Introduction

Polymer electrolyte fuel cells (PEFCs) have attracted much interest due to the need for an efficient, non-polluting power source with high energy density for vehicles in urban environments, as well as portable electronics [1,2]. Hydrogen is the most suitable fuel for a fuel cell powered vehicle, providing the highest conversion efficiency for fuel-on-board-to-electric-power, and generating zero polluting emission, since water is the only product of the hydrogen/air fuel cell process. The widespread utilization of technologies based on polymer electrolyte fuel cells (PEFCs) relies on the development of efficient, durable and economic materials. 

Significant efforts have been made in recent decades to investigate the direct electrochemical oxidation of alcohol and hydrocarbon fuels [3,4]. Among the liquid organic fuels, methanol has promising characteristics in terms of reactivity at low temperatures, storage, and handling [3]. Nevertheless, before this technology can reach large-scale application, some drawbacks related to poor electrochemical performance, the high cost of fuel cell components, long-term stability, etc. need be solved.

In a PEFC system, high costs derive from the use of noble metal catalysts, perfluorosulfonic acid polymer electrolyte membranes, bipolar plates, and auxiliary components [5]. Therefore, the development of cost-effective and high-performing polymer electrolyte membranes, enhanced electro-catalysts, and cheap bipolar plates that satisfy the target requirements of high performance and durability, represents a significant challenge. Research is currently being addressed towards development of cost-effective materials, such as novel hydrocarbon membranes [6,7] and low precious metal loading electrodes [8,9]. The key to a sustainable energy future thus lies in the development of advanced materials.

This Special Issue is intended to cover the most recent progress in advanced electro-catalysts, catalytic supports, electrodes, membranes, fillers and bipolar plates for high-performance and cost-effective polymer electrolyte fuel cells, including direct alcohol fuel cells.

## 2. This Special Issue

**Figure 1:**
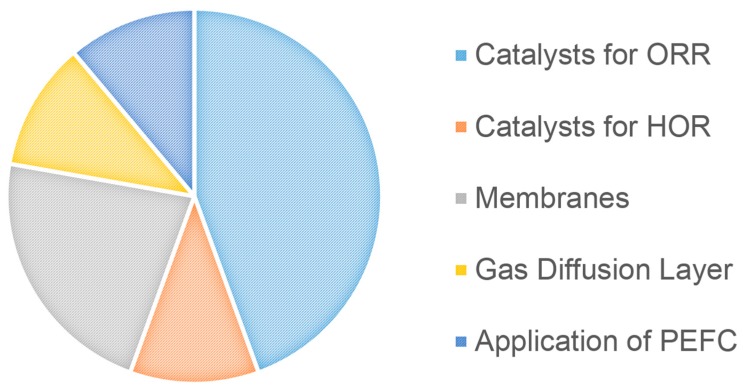
Distribution of the contributions of this Special Issue by topics.

This Special Issue covers recent work related to advances in materials development for low-temperature fuel cells; in particular, the design, synthesis, characterization and performance evaluation of catalysts, membranes, gas diffusion layers, and applications. It includes one review paper, and 8 original research articles. The contributions to this Special Issue can be distributed into the 5 topics indicated in the pie chart shown in Figure 1:

Five of the contributions relate to the investigation of catalytic materials for either the oxygen reduction reaction (ORR) or the hydrogen oxidation reaction (HOR), given that ORR is still one of the most important topics among research activities related to PEFCs. The activities related to membranes also occupy a relevant position in this issue, and are covered by a review paper and a research article. Research into materials for the gas diffusion layer in the electrodes and the potential application of PEFCs for separation are also covered in this Special Issue.

Research into catalysts based on formulations free of platinum group metals (PGMs) is gaining increasing interest within the scientific community due to the attractive possibility of substituting expensive precious metals with more economical, available, and green catalytic materials [9]. Yu et al. reported the synthesis of nitrogen-doped mesoporous carbon for the ORR [10]. Their investigation covers the effect of a set of synthesis conditions of interest, and how these influence the physico-chemical features of nitrogen-doped carbons, together with the evaluation of ORR activity in an alkaline environment using a rotating disk electrode. They concluded that carbon with two-dimensional morphology exhibits increased accessibility of active sites compared to three-dimensional carbon particles. Mittermeier et al. published an investigation of various transition-metal silicides as catalysts for the hydrogen oxidation and evolution reactions in acidic environments [11]. This is the first work that demonstrates sufficient chemical and electrochemical stability of metal silicides with Mo, W, Ta, Ni and Mo-Ni. 

On the other hand, there is still much interest in optimizing the utilization of PGM catalysts. Zignani et al. presented a study of the influence of carbon-supported PtNi nanoparticles as cathode catalysts for PEFCs under automotive conditions [12]. They concluded that the best Pt:Ni formulation in terms of activity (Pt_3_Ni_2_) differs from the best in terms of stability (Pt_1_Ni_1_). This indicates that a tradeoff situation in the use of ad-metals is necessary for balancing a good initial performance and appropriate durability of the device. Alegre et al. investigated the effect of nitrogen doping of advanced carbon materials (xerogels) on the activity and stability of Pt nanoparticles [13]. The doping of carbon supports represents another alternative for enhancing durability issues, as demonstrated in this publication, even though a careful selection of synthesis properties is necessary. As an alternative to platinum, palladium has also been proposed for the oxygen reduction reaction [14]. In this Special Issue, Rivera et al. reported the utilization of carbon-supported Pd and PdFe catalysts for the cathode of direct methanol fuel cells [15]. They showed that Pd is a promising substitute for Pt in direct methanol fuel cells due to an enhanced tolerance to crossovered methanol. They also concluded that alloying Pd with Fe yields structural changes to the noble metal crystal structure that further contributes to improve the fuel cell performance.

The research activities related to the development of polymer materials to be applied as electrolyte membranes is another hot topic in the field of PEFCs. Quartarone et al. reviewed, in this Special Issue, the state of the art of membranes fabrication and properties from a critical point of view [16]. In particular, they focused their attention on membranes for high-temperature PEFC application, which are composed of polybenzimidazole (PBI), including acid-based blends and composite membranes, and other polymers and co-polymers like sulphonated poly(ether ether ketone) (SPEEK). They concluded that although great advances have been reported for alternative membranes, PBI-based ones are still the best choice for high-temperature operation (150 °C–200 °C). Rangel-Cárdenas et al. described the transport properties of proton exchange membranes, proving that interface effects must be taken into account [17]. The approach consisted of evaluating transport properties in homogeneous membranes and also for a membrane with two surface layers. This method allowed for a critical evaluation of the literature values, and for the optimization of stacked transport systems. 

Other than catalyst and membrane materials, the gas diffusion layer of PEFCs plays a relevant role in performance. Jayakumar et al. reported in this Special Issue a novel technique for the manufacturing of gas diffusion layers [18]. They proposed an advanced 3D printing technique based on selective laser sintering for the first time. The aim was to solve some of the most relevant issues concerning gas diffusion layers, like electrochemical oxidation or mechanical degradation. Among other topics related to PEFC materials, Matsushima et al. demonstrated that PEFCs can serve to separate isotopes, in particular deuterium [19]. They concluded that the separation efficiency in this kind of system relies on the concentration of deuterium in the source gas.
